# Intestinal Dysbiosis and Autoimmune Pancreatitis

**DOI:** 10.3389/fimmu.2021.621532

**Published:** 2021-03-23

**Authors:** Tomoe Yoshikawa, Tomohiro Watanabe, Ken Kamata, Akane Hara, Kosuke Minaga, Masatoshi Kudo

**Affiliations:** Department of Gastroenterology and Hepatology, Kindai University, Faculty of Medicine, Ohno-Higashi, Osaka-Sayama, Japan

**Keywords:** autoimmune pancreatitis, dysbiosis, IgG4-related disease, plasmacytoid dendritic cells, intestinal microbiota

## Abstract

Autoimmune pancreatitis (AIP) is a chronic fibro-inflammatory disorder of the pancreas. Recent clinicopathological analysis revealed that most cases of AIP are pancreatic manifestations of systemic IgG4-related disease (IgG4-RD), a newly established disease characterized by enhanced IgG4 antibody responses and the involvement of multiple organs. Although the immuno-pathogenesis of AIP and IgG4-RD has been poorly defined, we recently showed that activation of plasmacytoid dendritic cells (pDCs) with the ability to produce large amounts of IFN-α and IL-33 mediates chronic fibro-inflammatory responses in experimental and human AIP. Moreover, M2 macrophages producing a large amount of IL-33 play pathogenic roles in the development of human IgG4-RD. Interestingly, recent studies including ours provide evidence that compositional alterations of gut microbiota are associated with the development of human AIP and IgG4-RD. In addition, intestinal dysbiosis plays pathological roles in the development of chronic pancreatic inflammation as dysbiosis mediates the activation of pDCs producing IFN-α and IL-33, thereby causing experimental AIP. In this Mini Review, we focus on compositional alterations of gut microbiota in AIP and IgG4-RD to clarify the mechanisms by which intestinal dysbiosis contributes to the development of these disorders.

## Introduction

Intestinal bacteria residing in the human gastrointestinal (GI) tract are essential components for the development of mucosal immune system, facilitation of digestion and absorption of food, and modulation of glucose metabolism (1–3). Microbial communities in the GI tract are composed of more than 10^14^ microorganisms and live symbiotically with the host (1–3). It is now generally accepted that compositional and functional alterations of the gut microbiome also known as intestinal dysbiosis, are involved in the development of GI tract diseases as shown by the well-established relationship between intestinal dysbiosis and inflammatory bowel disease (IBD) (1–3). In fact, excessive pro-inflammatory cytokine responses against intestinal microbiota underlie the immuno-pathogenesis of IBD (4, 5). It should be noted, however, that intestinal dysbiosis plays pathogenic roles in the development of not only GI tract diseases but also those affecting parts other than the GI tract. In line with this concept, accumulating evidence suggests possible involvement of intestinal dysbiosis in the development of several pancreatic diseases such as acute pancreatitis, chronic pancreatitis (CP), and pancreatic cancer (6–10).

Autoimmune pancreatitis (AIP) and CP are two major forms of chronic fibro-inflammatory disorders of the pancreas. CP is caused by frequent episodic activation of intrapancreatic digestive enzymes (9). Notably, environmental factors including excessive consumption of alcohol and smoking increase the risk of CP (9). Clinicopathological analyses revealed that AIP is a pancreatic manifestation of systemic IgG4-related disease (IgG4-RD), a new disease characterized by elevated concentrations of serum IgG4 antibody (Ab), accumulation of IgG4-expressing plasma cells into the affected organs, and involvement of multiple organs (11–14). Recent identification of candidate autoantigens in AIP and IgG4-RD support the concept that AIP and IgG4-RD are driven by autoimmune responses (15–17). Although enhanced IgG4 Ab responses are a hallmark of AIP and IgG4-RD, it remains unknown whether this IgG subtype plays pathogenic roles in these disorders. Shiokawa et al. directly addressed this issue by passively transferring patient IgG subtypes into neonatal mice and found that IgG1 Ab rather than IgG4 Ab has pathogenicity which drives chronic inflammation in AIP and IgG4-RD (18). Thus, enhanced IgG4 Ab responses seen in AIP and IgG4-RD are considered as epiphenomenon reflecting chronic inflammation. This notion is fully supported by the fact that IgG4 Ab exhibits poor ability to activate the complement system and Fc-γ receptors (19).

Considering that intestinal dysbiosis is observed in patients with autoimmune diseases (1–3, 20), excessive innate immune responses against intestinal microflora are likely to be involved in the development of AIP and IgG4-RD. However, little has been understood regarding the molecular mechanisms of intestinal dysbiosis and how they induce chronic fibro-inflammatory responses in the pancreas of AIP patients. Recently, we found that intestinal dysbiosis causes chronic fibro-inflammatory responses in the pancreas through activation of plasmacytoid dendritic cells (pDCs) with the ability to produce a large amount of IFN-α and IL-33 (21, 22). In this Mini-review article, we discuss the relationship between intestinal dysbiosis and AIP.

## Innate Immune Cytokines in AIP

Innate immunity is an initial component of the immune system involved in the eradication of invading microbial pathogens (23, 24). Toll-like receptors (TLRs) and nucleotide-binding oligomerization domain (NOD)-like receptors (NLRs) are innate immune receptors which recognize microbe-associated molecular patterns (MAMPs) and induce pro-inflammatory cytokine responses for host defense against invading microbes (23, 24). Although AIP and IgG4-RD are characterized by enhanced IgG4 Ab production, i.e., adaptive immunity, recent studies highlight the importance of innate immunity as shown by enhanced expression of TLRs in the pancreas and salivary glands of patients with AIP and IgG4-RD (25, 26). Moreover, the roles played by TLR7 expressed in M2 macrophages have been particularly implicated in the pathogenesis of AIP and IgG4-RD (25, 27).

Activation of TLRs and NLRs in antigen-presenting cells (APCs) such as macrophages and dendritic cells results in pro-inflammatory cytokine responses through activation of transcription factors (23, 24). If innate immune responses against intestinal microbiota are involved in the development of AIP, then which types of cytokines cause chronic fibro-inflammatory responses in the pancreas? To identify pathogenic cytokines and APCs, we utilized a well-established model of experimental AIP (28, 29). Repeated intraperitoneal injection of polyinosinic-polycytidylic acid (poly (I:C)), a synthetic TLR3 ligand, into MRL/MpJ mice led to the generation of pancreatic chronic fibro-inflammatory responses characterized by destruction of acinar architecture, immune cell infiltration, and fibrosis (30). Although this experimental AIP model shares pathologic findings with human AIP, its molecular mechanisms have been poorly defined. To explore pathogenic APC populations responsible for the development of experimental AIP, we determined alterations in the percentages of innate immune cells which accumulated in the pancreas of MRL/MpJ mice (28). Interestingly, the pancreas of AIP mice was characterized by a marked increase in the number of pDCs, defined as pDC antigen-1^+^B220^low^ cells by flow-cytometric analysis, as compared with that of control non-treated mice (28). pDCs are a specialized DC population with the ability to produce a large amount of IFN-α upon recognition of MAMPs by TLRs (31, 32). Activation of pDCs followed by a robust production of IFN-α plays critical roles in the development of experimental AIP since systemic administration of pDCs-depleting Ab or type I IFN receptor neutralizing Ab almost completely prevented the development of AIP (28).

**Figure 1:**
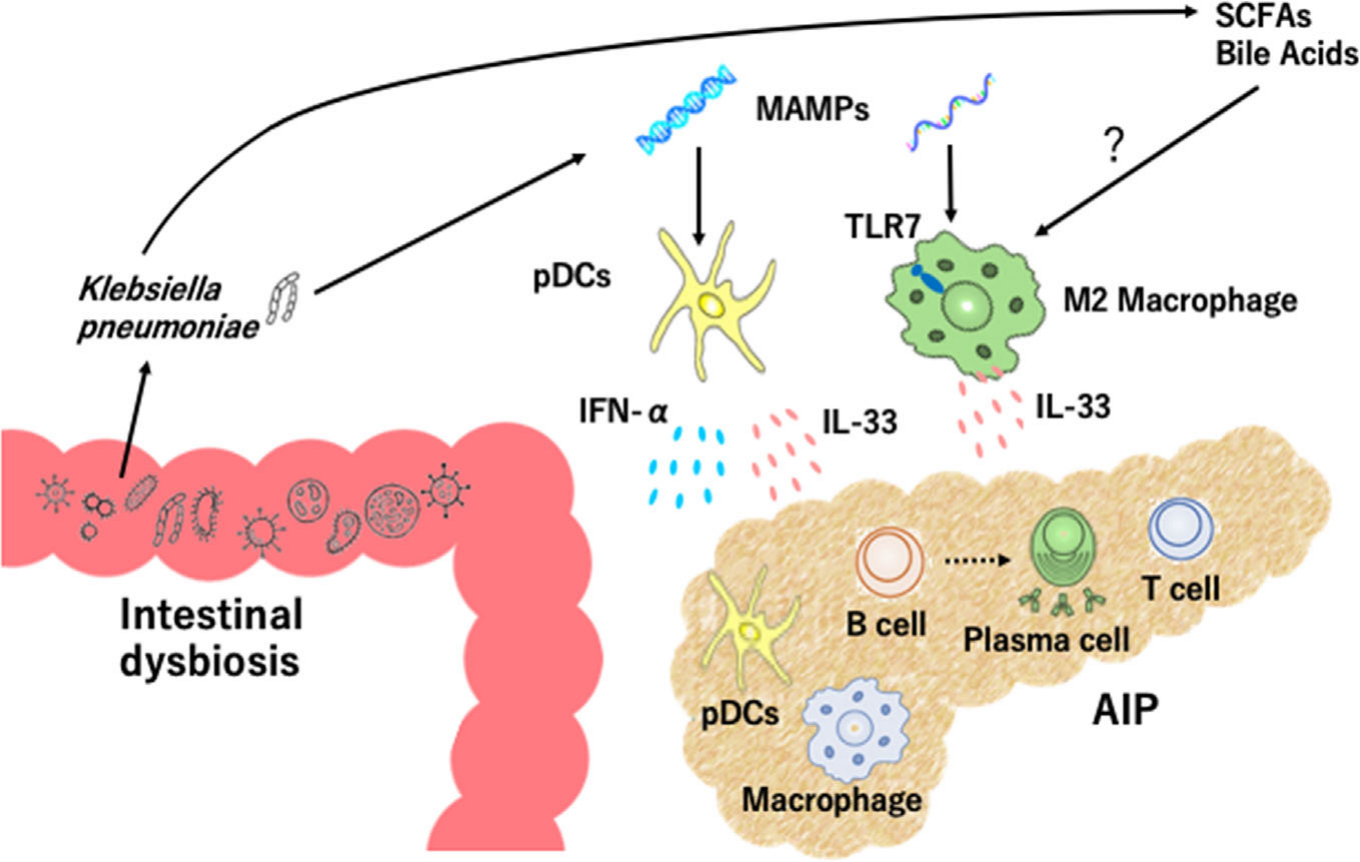
Intestinal dysbiosis and autoimmune pancreatitis. Intestinal dysbiosis activates plasmacytoid dendritic cells (pDCs) which produce IFN-α and IL-33. *Klebsiella pneumoniae* and microbe-associated molecular patterns (MAMPs) activate pancreatic pDCs to produce IFN-α and IL-33. Recognition of MAMPs by toll-like receptor 7 (TLR7) and exposure to short-chain fatty acids (SCFAs) and bile acids may lead to IL-33 production by M2 macrophages. Accumulation of pDCs and M2 macrophages in the pancreas causes infiltration of immune cells including IgG4-expressing plasmacytes, B cells, and T cells, destruction of acinar architecture, and fibrosis.

A specific form of fibrosis, called storiform fibrosis, is one of the characteristic pathological findings in AIP and IgG4-RD. In the case of experimental CP, type I IFN responses cause profibrogenic IL-33 production by pancreatic acinar cells (33). These findings obtained from an experimental CP model led us to examine roles played by IL-33 in experimental AIP. Pancreatic expression of IL-33 was much higher in MRL/MpJ mice treated with repeated injections of poly (I:C) than in non-treated mice (29). Depletion and purification studies using pancreatic mononuclear cells showed that pDCs are a cellular source of IL-33 and that pDCs produce this cytokine in a type I IFN-dependent manner (29). Neutralization of IL-33-mediated signaling pathways by ant-ST2 Ab attenuated chronic fibro-inflammatory responses in mice treated with poly (I:C) (29). These data support the view that activation of pDCs producing both IFN-α and IL-33 underlie the immuno-pathogenesis of experimental AIP (**Figure 1**).

Clinical relevance of the above findings obtained in murine experimental models of AIP was tested in human clinical samples. pDCs expressing IFN-α and/or IL-33 accumulate in the pancreas of patients with IgG4-related AIP, but not chronic alcoholic pancreatitis or non-cancerous portions of pancreatic cancer (28, 29, 34). In addition, peripheral blood pDCs isolated from patients with IgG4-related AIP efficiently induced IgG4 Ab production upon co-culture with healthy control B cells as compared with healthy control pDCs (28, 29). Taken together, these results strongly suggest that activation of pDCs and robust production of IFN-α and IL-33 are prominent features of murine experimental and human AIP. This idea has been fully supported by recent identification of serum IFN-α and IL-33 as novel biomarkers for human AIP and IgG4-RD (35). Although recognition of MAMPs by TLR7 or TLR9 in pDCs induces IFN-α production (31, 32), the involvement of these TLRs in pDCs needs to be determined (**Figure 1**).

The cellular source of IL-33 is not limited to pDCs alone since M2 macrophages expressing IL-33 are localized in the salivary glands of patients with IgG4-RD (27). Dual immunofluorescence analyses clearly showed that IL-33 was colocalized with M2 macrophages expressing CD68 or CD163 in the salivary glands of patients with IgG4-RD (27). Importantly, TLR7 expression was significantly higher in the salivary glands of patients with IgG4-RD than those with Sjogren syndrome or healthy controls (25, 27). CD163^+^ M2 macrophages isolated from patients with IgG4-RD produced a large amount of IL-33 upon stimulation with TLR7 ligands (25, 27). Furthermore, transgenic mice expressing the human TLR7 displayed autoimmune sialadenitis and pancreatitis which were accompanied by enhanced IL-33 production. These data imply that TLR7 activation in M2 macrophages mediate the development of IgG4-RD through IL-33 production (**Figure 1**).

## Intestinal Dysbiosis in Experimental AIP

Recognition of MAMPs derived from intestinal microbiota by TLRs induces IFN-α production by pDCs (31, 32). Thus, intestinal dysbiosis might be one of the possible triggers for the development of AIP and IgG4-RD. To determine the roles played by immune responses against intestinal microbiota in the development of experimental AIP, MRL/MpJ mice were treated with a broad range of antibiotics in their drinking water in combination with repeated injections of poly (I:C) (21). Bowel sterilization by antibiotic treatment inhibited the development of experimental AIP induced by poly (I:C) and resulted in a marked reduction in pancreatic accumulation of pDCs producing IFN-α and IL-33 (21). Severity of AIP in MRL/MpJ mice depends upon the doses of poly (I:C); repeated injections of 100 μg and 10 μg of poly (I:C) induces severe and mild types of AIP, respectively (21). This relationship between poly (I:C) doses and AIP severity enabled us to address whether intestinal dysbiosis alters sensitivity to experimental AIP through co-housing and fecal microbiota transplantation (FMT) studies. In the co-housing and FMT experiments to evaluate the effects of transmission of intestinal microbiota, we found that transmission of intestinal microflora from severe AIP mice treated with 100 μg of poly (I:C) altered the disease severity of mice treated with 10 μg of poly (I:C). Transmission of intestinal microflora from mice with severe AIP promoted pancreatic accumulation of pDCs producing IFN-α and IL-33 (21). Taken together, these studies provide evidence that intestinal dysbiosis mediates experimental AIP through activation of pDCs producing IFN-α and IL-33 (**Figure 1**).

## Intestinal Dysbiosis in Human AIP

Clinical relevance of intestinal dysbiosis was examined in human fecal samples from patients with IgG4-associated AIP. Gut microbiota profiles are different between patients with AIP and CP and the proportions of *Bacteroides*, *Streptococcus*, and *Clostridium* species were higher in the latter disease (36). Oral administration of prednisolone (PSL) induces clinical remission in most cases of AIP and IgG4-RD (11–14). We initially addressed alterations in fecal microbiota composition in three patients with IgG4-associated AIP (22). Although a variety of alterations in fecal microbiota were seen after induction of remission in these three patients, the most striking finding was the complete disappearance of *Klebsiella pneumoniae* (*K. pneumoniae*) from the feces in two of three patients. Thus, induction of remission might be associated with disappearance of *K. pneumoniae* in patients with IgG4-associated AIP. To determine pathogenicity of *K. pneumoniae* in AIP, MRL/MpJ mice were orally treated with heat-killed *K. pneumoniae* in combination with an injection of 10 μg of poly (I:C). We found that the severity of experimental AIP was greater in mice treated with oral administration of *K. pneumoniae* and injection of 10 μg of poly (I:C) than in those with either treatment alone. Moreover, the development of severe AIP in mice that received both treatments was accompanied by pancreatic accumulation of pDCs producing IFN-α and IL-33 (22). These data strongly suggest that gut colonization of *K. pneumoniae* increases the sensitivity of AIP through activation of pDCs which produce IFN-α and IL-33. Pathogenicity of *K. pneumoniae* was also reported in a previous report showing that immunization with a mixure of extract of pooled pancreas from syngeneic mice and the capsular polysaccharide of *K. pneumoniae* led to the development of chronic fibro-inflammatory responses in the pancreas (37). Collectively, these studies strongly support involvement of intestinal dysbiosis in the development of AIP. It should be emphasized that intestinal dysbiosis enhances the sensitivity to AIP through the activation of pDCs, but cannot cause AIP on its own. Thus, intestinal dysbiosis functions as a disease intensifier rather than a primary pathogenic factor in the development of AIP (**Figure 1**).

As previously mentioned, M2 macrophages which express TLR7 contribute to the development of AIP and IgG4-RD (25, 27). However, it remains unknown whether intestinal dysbiosis seen in AIP promotes IL-33 production by M2 macrophages. Recognition of MAMPs by TLR7 in the presence of dysbiosis might be involved in the development of IgG4-RD and AIP.

Molecular mechanisms involved in the activation of pDCs and M2 macrophages by intestinal dysbiosis have been poorly defined. In this regard, two possibilities have been considered: the first one is that intestinal dysbiosis activates pDCs or M2 macrophages residing in the GI tract and then these cells migrate to the pancreas. The second possibility is that pDCs or M2 macrophages in the pancreas are directly activated by intestinal bacteria translocated into this organ. We think that the latter is more likely because pancreatic pDCs isolated from mice exhibiting AIP produced large amounts of IFN-α and IL-33 upon stimulation with *K. pneumoniae* (22). Moreover, the fact that pDCs from the Peyer’s patches are unable to produce IFN-α upon stimulation with TLR7 and TLR9 ligands supports the second option (38).

## Intestinal Dysbiosis and Adaptive Immunity Associated With AIP

A wide varieties of T cell subpopulations including T helper type 2 (Th2) cells, regulatory T cells (Tregs), and follicular helper T cells have been identified in the peripheral blood and affected organs in AIP and IgG4-RD (12). Haruta et al. developed a unique model of murine experimental AIP caused by repeated exposures to *Escherichia coli* (39). Splenocytes isolated from mice inoculated with *E. coli* efficiently induced AIP in RAG2-deficient mice upon adaptive transfer, suggesting that the development of this unique AIP requires adaptive immune responses (39). Although the relationship between intestinal dysbiosis and effector CD4^+^ T cell responses has not been elucidated, pDCs or M2 macrophages may be involved in the generation of effector T cell responses. Given that IL-33 is a well-established activator of Th2 responses (40), IL-33 produced by pDCs or M2 macrophages in response to intestinal dysbiosis may promote differentiation of Th2 cells (21, 25, 27, 29, 34). Moreover, pDCs enhance proliferation of forkhead box P3 (Foxp3)^+^ Tregs (31, 41). Therefore, it is plausible that intestinal dysbiosis mediates effector T cell responses through the interaction with pDCs and M2 macrophages. In addition, MAMPs derived from intestinal bacteria have been reported to be potent stimulators of IgG4 Ab class-switch recombination in a co-culture system composed of monocytes and naïve B cells (42), suggesting that exposure to intestinal microbiota can augment IgG4 Ab responses characterizing AIP and IgG4-RD. Verification of this idea awaits future studies.

Recently, candidate auto-antigens have been successfully identified in AIP and IgG4-RD (15–17). It would be intriguing to determine the effects of intestinal dysbiosis on adaptive immunity specific to pathogenic antigens. The molecular mimicry between intestinal bacteria and these autoantigens can be a trigger for the generation of pathogenic adaptive immune responses. Alternatively, cytokines including IFN-α and IL-33 augment pathogenic antigen-specific immune responses.

## Microbial Metabolites and AIP

Intestinal microbiota engage in diverse metabolic processes including fermentation of amino acids, generation of vitamins, and modification of bile acids (2, 43). Although the effects of microbial metabolites on immune responses associated with AIP have not been studied, intestinal dysbiosis may result in alterations of microbial metabolites. Short-chain fatty acids (SCFAs) are produced by microbiota after fermentation of dietary fibers and have been shown to facilitate the polarization and function of M2 macrophages (44). Bile acids activate the transmembrane G protein-coupled receptor 5 (TGR5) and farnesoid X receptor (FXR) expressed in macrophages. Activation of TGR5 and FXR leads to M2 macrophage differentiation (45, 46). Investigating whether SCFAs and bile acids promote IL-33 production by M2 macrophages is an interesting research question. Given that SCFAs efficiently induce differentiation of Tregs (47), SCFAs and bile acids may act together to generate immune environments causing AIP and IgG4-RD. It should be noted, however, that future studies must be done to investigate alterations in microbial metabolites in AIP.

## Conclusions

Recent studies using experimental models of AIP highlight the importance of intestinal dysbiosis in the development of chronic fibro-inflammatory responses in the pancreas. Alterations in gut microbiota composition may function as a disease intensifier rather than a direct pathogenic factor through activation of pDCs which produce IFN-α and IL-33 and M2 macrophages which express TLR7. It should be noted, however, that recent data regarding fecal microbiota composition in human AIP were obtained by using a limited number of patients (22, 36). Thus, fecal microbiota analyses using a large number of AIP patients are absolutely required. Moreover, molecular mechanisms of AIP induction *via* the gut-pancreas axis have been poorly defined. The sites in which pathogenic immune responses are generated have not been clarified. It remains largely unknown whether intestinal bacteria translocating to the pancreas activate *in situ* immune responses or gut immune cells activated by intestinal dysbiosis migrate to the pancreas to cause pancreatitis? Intestinal bacteria with the ability to promote or inhibit pancreatic inflammation have not been identified. Further studies are necessary to establish the link between intestinal dysbiosis and murine and human AIP.

## Author Contributions

TY and TW wrote the manuscript draft. KK, AH, KM, and MK revised and edited the manuscript. All authors contributed to the article and approved the submitted version.

## Funding

This work was supported by Grants-in-Aid for Scientific Research (19K08455, 19K17506, 20K16975) from the Japan Society for the Promotion of Science, Takeda Science Foundation, Smoking Research Foundation, Yakult Bio-Science Foundation, SENSHIN Medical Research Foundation, and Japan Agency for Medical Research and Development (AMED) for Research on Intractable Diseases.

## Conflict of Interest

The authors declare that the research was conducted in the absence of any commercial or financial relationships that could be construed as a potential conflict of interest.

## References

[1] Levy M Kolodziejczyk AA Thaiss CA Elinav E. Dysbiosis and the immune system. Nat Rev Immunol (2017) 17:219–32. 10.1038/nri.2017.7 28260787

[2] Skelly AN Sato Y Kearney S Honda K. Mining the microbiota for microbial and metabolite-based immunotherapies. Nat Rev Immunol (2019) 19:305–23. 10.1038/s41577-019-0144-5 30858494

[3] Lynch SV Pedersen O. The Human Intestinal Microbiome in Health and Disease. N Engl J Med (2016) 375:2369–79. 10.1056/NEJMra1600266 27974040

[4] Honjo H Watanabe T Arai Y Kamata K Minaga K Komeda Y. ATG16L1 negatively regulates RICK/RIP2-mediated innate immune responses. Int Immunol (2021) 33:91–105. 10.1093/intimm/dxaa062 32909611

[5] Watanabe T Minaga K Kamata K Sakurai T Komeda Y Nagai T. RICK/RIP2 is a NOD2-independent nodal point of gut inflammation. Int Immunol (2019) 31:669–83. 10.1093/intimm/dxz045 PMC6939834 31132297

[6] Adolph TE Mayr L Grabherr F Schwarzler J Tilg H. Pancreas-Microbiota Cross Talk in Health and Disease. Annu Rev Nutr (2019) 39:249–66. 10.1146/annurev-nutr-082018-124306 31433743

[7] Akshintala VS Talukdar R Singh VK Goggins M. The Gut Microbiome in Pancreatic Disease. Clin Gastroenterol Hepatol (2019) 17:290–5. 10.1016/j.cgh.2018.08.045 PMC6314887 30144522

[8] Thomas RM Jobin C. Microbiota in pancreatic health and disease: the next frontier in microbiome research. Nat Rev Gastroenterol Hepatol (2020) 17:53–64. 10.1038/s41575-019-0242-7 31811279

[9] Watanabe T Kudo M Strober W. Immunopathogenesis of pancreatitis. Mucosal Immunol (2017) 10:283–98. 10.1038/mi.2016.101 27848953

[10] Tsuji Y Watanabe T Kudo M Arai H Strober W Chiba T. Sensing of Commensal Organisms by the Intracellular Sensor NOD1 Mediates Experimental Pancreatitis. Immunity (2012) 37:326–38. 10.1016/j.immuni.2012.05.024 PMC3523885 22902233

[11] Kamisawa T Chari ST Lerch MM Kim MH Gress TM Shimosegawa T. Recent advances in autoimmune pancreatitis: type 1 and type 2. Gut (2013) 62:1373–80. 10.1136/gutjnl-2012-304224 23749606

[12] Watanabe T Minaga K Kamata K Kudo M Strober W. Mechanistic Insights into Autoimmune Pancreatitis and IgG4-Related Disease. Trends Immunol (2018) 39:874–89. 10.1016/j.it.2018.09.005 30401468

[13] Kamisawa T Zen Y Pillai S Stone JH. IgG4-related disease. Lancet (2015) 385:1460–71. 10.1016/S0140-6736(14)60720-0 25481618

[14] Stone JH Zen Y Deshpande V. IgG4-related disease. N Engl J Med (2012) 366:539–51. 10.1056/NEJMra1104650 22316447

[15] Shiokawa M Kodama Y Sekiguchi K Kuwada T Tomono T Kuriyama K. Laminin 511 is a target antigen in autoimmune pancreatitis. Sci Trans Med (2018) 10:eaaq0997. 10.1126/scitranslmed.aaq0997 30089633

[16] Perugino CA AlSalem SB Mattoo H Della-Torre E Mahajan V Ganesh G. Identification of galectin-3 as an autoantigen in patients with IgG4-related disease. J Allergy Clin Immunol (2019) 143:736–45.e6. 10.1016/j.jaci.2018.05.011 29852256 PMC6265117

[17] Hubers LM Vos H Schuurman AR Erken R Oude Elferink RP Burgering B. Annexin A11 is targeted by IgG4 and IgG1 autoantibodies in IgG4-related disease. Gut (2018) 67:728–35. 10.1136/gutjnl-2017-314548 28765476

[18] Shiokawa M Kodama Y Kuriyama K Yoshimura K Tomono T Morita T. Pathogenicity of IgG in patients with IgG4-related disease. Gut (2016) 65:1322–32. 10.1136/gutjnl-2015-310336 26964842

[19] Aalberse RC Stapel SO Schuurman J Rispens T. Immunoglobulin G4: an odd antibody. Clin Exp Allergy (2009) 39:469–77. 10.1111/j.1365-2222.2009.03207.x 19222496

[20] Bach JF. The hygiene hypothesis in autoimmunity: the role of pathogens and commensals. Nat Rev Immunol (2018) 18:105–20. 10.1038/nri.2017.111 29034905

[21] Kamata K Watanabe T Minaga K Hara A Yoshikawa T Okamoto A. Intestinal dysbiosis mediates experimental autoimmune pancreatitis via activation of plasmacytoid dendritic cells. Int Immunol (2019) 31:795–809. 10.1093/intimm/dxz050 31287532

[22] Kamata K Watanabe T Minaga K Hara A Sekai I Otsuka Y. Gut microbiome alterations in type 1 autoimmune pancreatitis after induction of remission by prednisolone. Clin Exp Immunol (2020) 202:308–20. 10.1111/cei.13509 PMC7670154 32880930

[23] Takeda K Akira S. Toll-like receptors in innate immunity. Int Immunol (2005) 17:1–14. 10.1093/intimm/dxh186 15585605

[24] Strober W Murray PJ Kitani A Watanabe T. Signalling pathways and molecular interactions of NOD1 and NOD2. Nat Rev Immunol (2006) 6:9–20. 10.1038/nri1747 16493424

[25] Ishiguro N Moriyama M Furusho K Furukawa S Shibata T Murakami Y. Activated M2 Macrophages Contribute to the Pathogenesis of IgG4-Related Disease via Toll-like Receptor 7/Interleukin-33 Signaling. Arthritis Rheumatol (2020) 72:166–78. 10.1002/art.41052 PMC6972995 31339007

[26] Fukui Y Uchida K Sakaguchi Y Fukui T Nishio A Shikata N. Possible involvement of Toll-like receptor 7 in the development of type 1 autoimmune pancreatitis. J Gastroenterol (2015) 50:435–44. 10.1007/s00535-014-0977-4 25005350

[27] Furukawa S Moriyama M Miyake K Nakashima H Tanaka A Maehara T. Interleukin-33 produced by M2 macrophages and other immune cells contributes to Th2 immune reaction of IgG4-related disease. Sci Rep (2017) 7:42413. 10.1038/srep42413 28205524 PMC5304322

[28] Arai Y Yamashita K Kuriyama K Shiokawa M Kodama Y Sakurai T. Plasmacytoid Dendritic Cell Activation and IFN-alpha Production Are Prominent Features of Murine Autoimmune Pancreatitis and Human IgG4-Related Autoimmune Pancreatitis. J Immunol (2015) 195:3033–44. 10.4049/jimmunol.1500971 26297761

[29] Watanabe T Yamashita K Arai Y Minaga K Kamata K Nagai T. Chronic Fibro-Inflammatory Responses in Autoimmune Pancreatitis Depend on IFN-alpha and IL-33 Produced by Plasmacytoid Dendritic Cells. J Immunol (2017) 198:3886–96. 10.4049/jimmunol.1700060 28373582

[30] Kamata K Watanabe T Minaga K Strober W Kudo M. Autoimmune Pancreatitis Mouse Model. Curr Protoc Immunol (2018) 120:15 31 1–15 31 8. 10.1002/cpim.41 29512140 PMC5880538

[31] Swiecki M Colonna M. The multifaceted biology of plasmacytoid dendritic cells. Nat Rev Immunol (2015) 15:471–85. 10.1038/nri3865 PMC4808588 26160613

[32] Ganguly D. Do Type I Interferons Link Systemic Autoimmunities and Metabolic Syndrome in a Pathogenetic Continuum? Trends Immunol (2018) 39:28–43. 10.1016/j.it.2017.07.001 28826817

[33] Watanabe T Sadakane Y Yagama N Sakurai T Ezoe H Kudo M. Nucleotide-binding oligomerization domain 1 acts in concert with the cholecystokinin receptor agonist, cerulein, to induce IL-33-dependent chronic pancreatitis. Mucosal Immunol (2016) 9:1234–49. 10.1038/mi.2015.144 26813347

[34] Minaga K Watanabe T Arai Y Shiokawa M Hara A Yoshikawa T. Activation of interferon regulatory factor 7 in plasmacytoid dendritic cells promotes experimental autoimmune pancreatitis. J Gastroenterol (2020) 55:565–76. 10.1007/s00535-020-01662-2 31960143

[35] Minaga K Watanabe T Hara A Kamata K Omoto S Nakai A. Identification of serum IFN-alpha and IL-33 as novel biomarkers for type 1 autoimmune pancreatitis and IgG4-related disease. Sci Rep (2020) 10:14879. 10.1038/s41598-020-71848-4 32938972 PMC7495433

[36] Hamada S Masamune A Nabeshima T Shimosegawa T. Differences in Gut Microbiota Profiles between Autoimmune Pancreatitis and Chronic Pancreatitis. Tohoku J Exp Med (2018) 244:113–7. 10.1620/tjem.244.113 29434076

[37] Yamaki K Ohta M Nakashima I Noda A Asai J Kato N. Microbial adjuvant and autoimmunity. IV. Production of lesions in the exocrine pancreas of mice by repeated injection of syngeneic pancreatic extract together with the capsular polysaccharide of Klebsiella pneumoniae. Microbiol Immunol (1980) 24:945–56. 10.1111/j.1348-0421.1980.tb02900.x 7007828

[38] Contractor N Louten J Kim L Biron CA Kelsall BL. Cutting edge: Peyer’s patch plasmacytoid dendritic cells (pDCs) produce low levels of type I interferons: possible role for IL-10, TGFbeta, and prostaglandin E2 in conditioning a unique mucosal pDC phenotype. J Immunol (2007) 179:2690–4. 10.4049/jimmunol.179.5.2690 17709480

[39] Haruta I Yanagisawa N Kawamura S Furukawa T Shimizu K Kato H. A mouse model of autoimmune pancreatitis with salivary gland involvement triggered by innate immunity via persistent exposure to avirulent bacteria. Lab Invest (2010) 90:1757–69. 10.1038/labinvest.2010.153 20733561

[40] Cayrol C Girard JP. IL-33: an alarmin cytokine with crucial roles in innate immunity, inflammation and allergy. Curr Opin Immunol (2014) 31C:31–7. 10.1016/j.coi.2014.09.004 25278425

[41] Conrad C Gregorio J Wang YH Ito T Meller S Hanabuchi S. Plasmacytoid dendritic cells promote immunosuppression in ovarian cancer via ICOS costimulation of Foxp3(+) T-regulatory cells. Cancer Res (2012) 72:5240–9. 10.1158/0008-5472.CAN-12-2271 PMC3652570 22850422

[42] Watanabe T Yamashita K Fujikawa S Sakurai T Kudo M Shiokawa M. Activation of Toll-like Receptors and NOD-like Receptors Is Involved in Enhanced IgG4 Responses in Autoimmune Pancreatitis. Arthritis Rheumatism (2012) 64:914–24. 10.1002/art.33386 21971969

[43] Garrett WS. Immune recognition of microbial metabolites. Nat Rev Immunol (2020) 20:91–2. 10.1038/s41577-019-0252-2 31768024

[44] Ji J Shu D Zheng M Wang J Luo C Wang Y. Microbial metabolite butyrate facilitates M2 macrophage polarization and function. Sci Rep (2016) 6:24838. 10.1038/srep24838 27094081 PMC4837405

[45] Biagioli M Carino A Cipriani S Francisci D Marchiano S Scarpelli P. The Bile Acid Receptor GPBAR1 Regulates the M1/M2 Phenotype of Intestinal Macrophages and Activation of GPBAR1 Rescues Mice from Murine Colitis. J Immunol (2017) 199:718–33. 10.4049/jimmunol.1700183 28607110

[46] Vavassori P Mencarelli A Renga B Distrutti E Fiorucci S. The bile acid receptor FXR is a modulator of intestinal innate immunity. J Immunol (2009) 183:6251–61. 10.4049/jimmunol.0803978 19864602

[47] Arpaia N Campbell C Fan X Dikiy S van der Veeken J deRoos P. Metabolites produced by commensal bacteria promote peripheral regulatory T-cell generation. Nature (2013) 504:451–5. 10.1038/nature12726 PMC3869884 24226773

